# Supplementary material for: Collagen density regulates the activity of tumor-infiltrating T cells
Source: J Immunother Cancer. 2019 Mar 12;7:68. doi: 10.1186/s40425-019-0556-6 (PMC6417085; doi:10.1186/s40425-019-0556-6)
Supplement: Supplementary file 1 — Figure S1. Proliferation of CDLA and CD8A T cells. Figure S2. Fraction of T cell subsets after HD culture or %D culture in different collagen densities. Figure S3. Analysis of CD8+ T cell abundance in areas of high and low collagen density in 20 triple negative breast cancer samples. Figure S4. RNAseq data. Figure S5. Regulated transcription factors after 2D culture or 3D culture in low density collagen. Table S2. Sequences of primers used for RT-qPCR. All primers were designed using the NCBI gene database and the primer-BLAST tool. Table S3. RNA sequencing depth and alignment info. (PDF 1041 kb) [file 40425_2019_556_MOESM1_ESM.pdf]

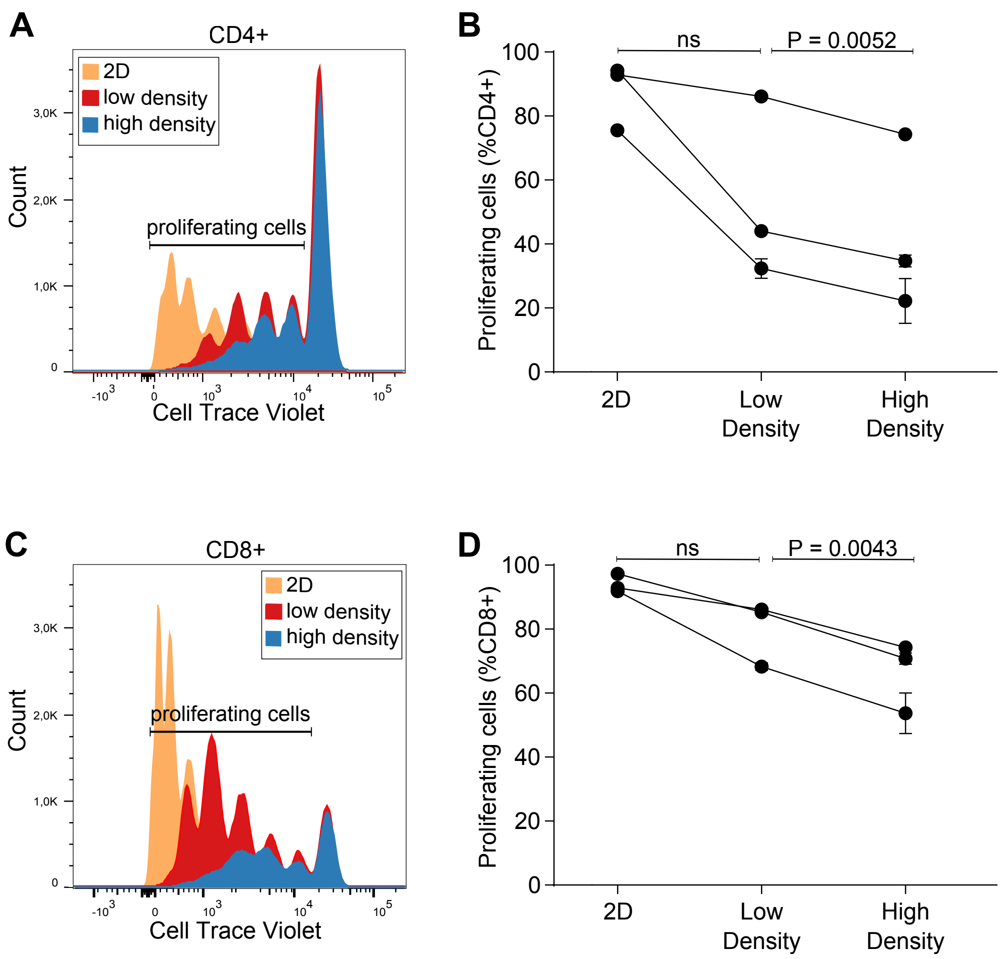

Figure S1. Proliferation of CD4+ and CD8+ T cells
Proliferation of CD4+ and CD8+ T cells after 5 days in culture was measured by flow cytometry based analysis of CellTrace Violet (CTV) dilution. (A and C) Representative histogram showing CTV dilution in CD4+ T cells (A) or CD8+ T cells (C) cultured in 2D or in 3D in a low-density collagen matrix or high-density collagen matrix. (B and D) Quantification of CD4+ T cell (B) or CD8+ T cell (D) proliferation based on CTV dilution. Three individual donors were analyzed. Connecting lines indicate measurements of the same donor.

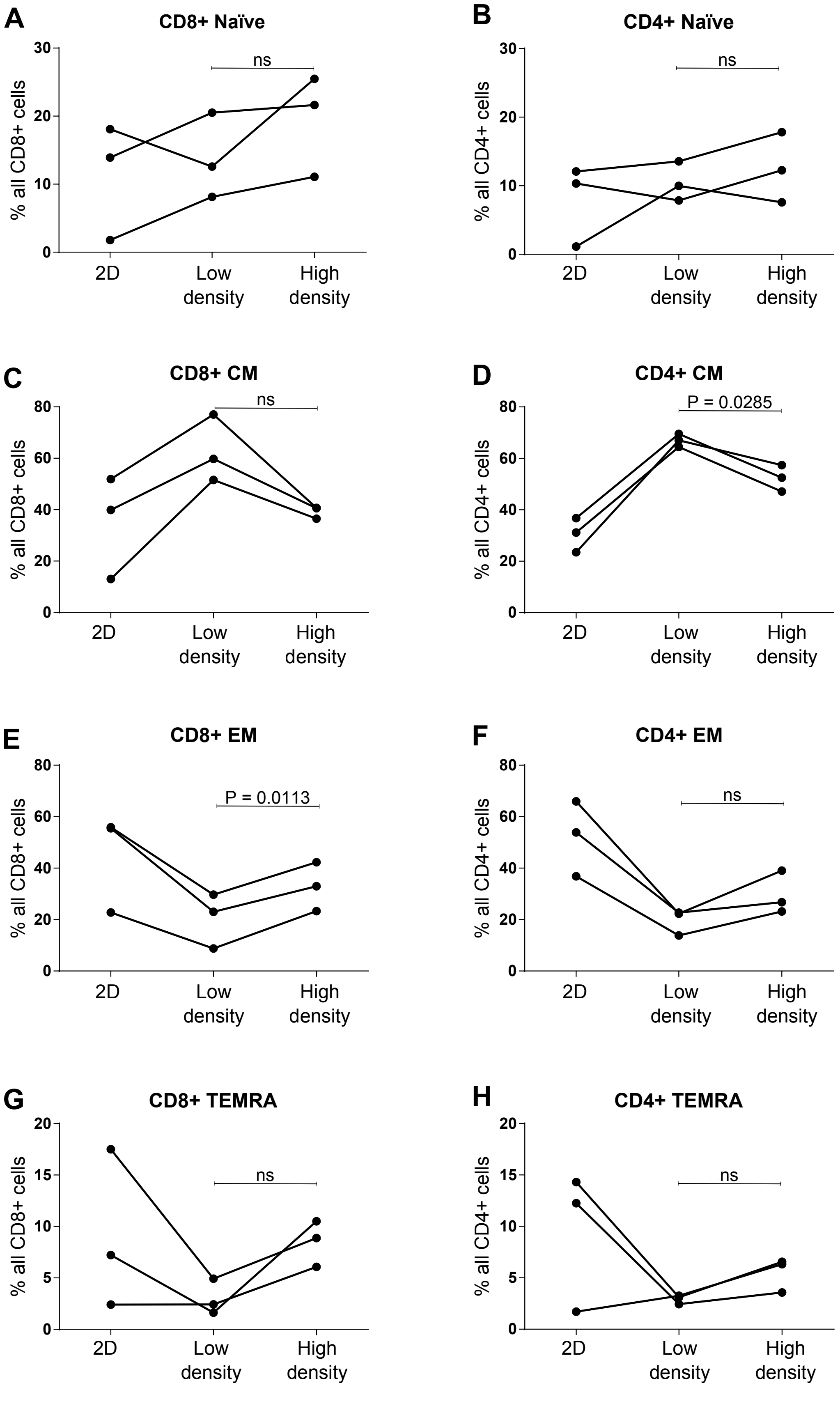

Figure S2. Fraction of T cell subsets after 2D culture or 3D culture in different collagen densities.

T cells were cultured for 5 days on plastic (2D) or in collagen matrices of high- or low density and analyzed by flow cytometry. CD8+ cells (A, C, E, G) and CD4+ cells (B, D, F, G) were divided into Naïve (CD45RA+,CD62L+ (A-B)), Central Memory (CD45RA-,CD62L+ (C -D)), Effector Memory (CD45RA-,CD62L- E-F)), or TEMRA cells (CD45RA+,CD62L- (G-H)). Three individual donors were analyzed. Connecting lines indicate measurements of the same donor.

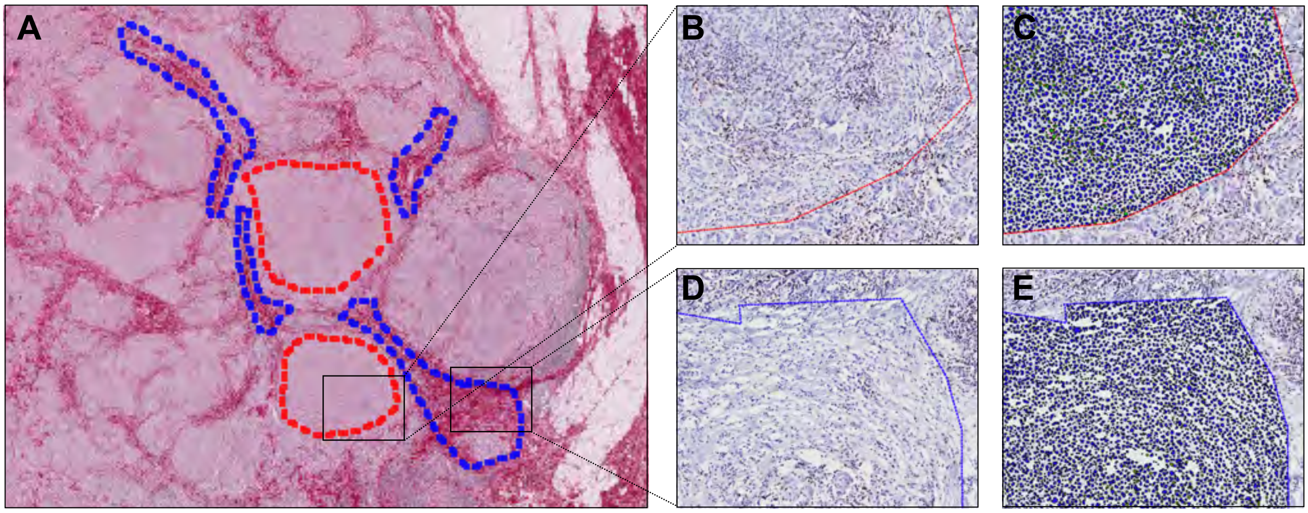

Figure S3. Analysis of CD8+ T cell abundance in areas of high and low collagen density in 20 triple negative breast cancer samples.

(A) Areas of high (blue dotted lines) and low (red dotted lines) collagen density were defined using the picrosirius red staining. (B-E) CD8 positive cells were detected in low (B-C) and high (D-E) collagen density areas defined in (A) after copying them to the CD8 stained adjacent sections.

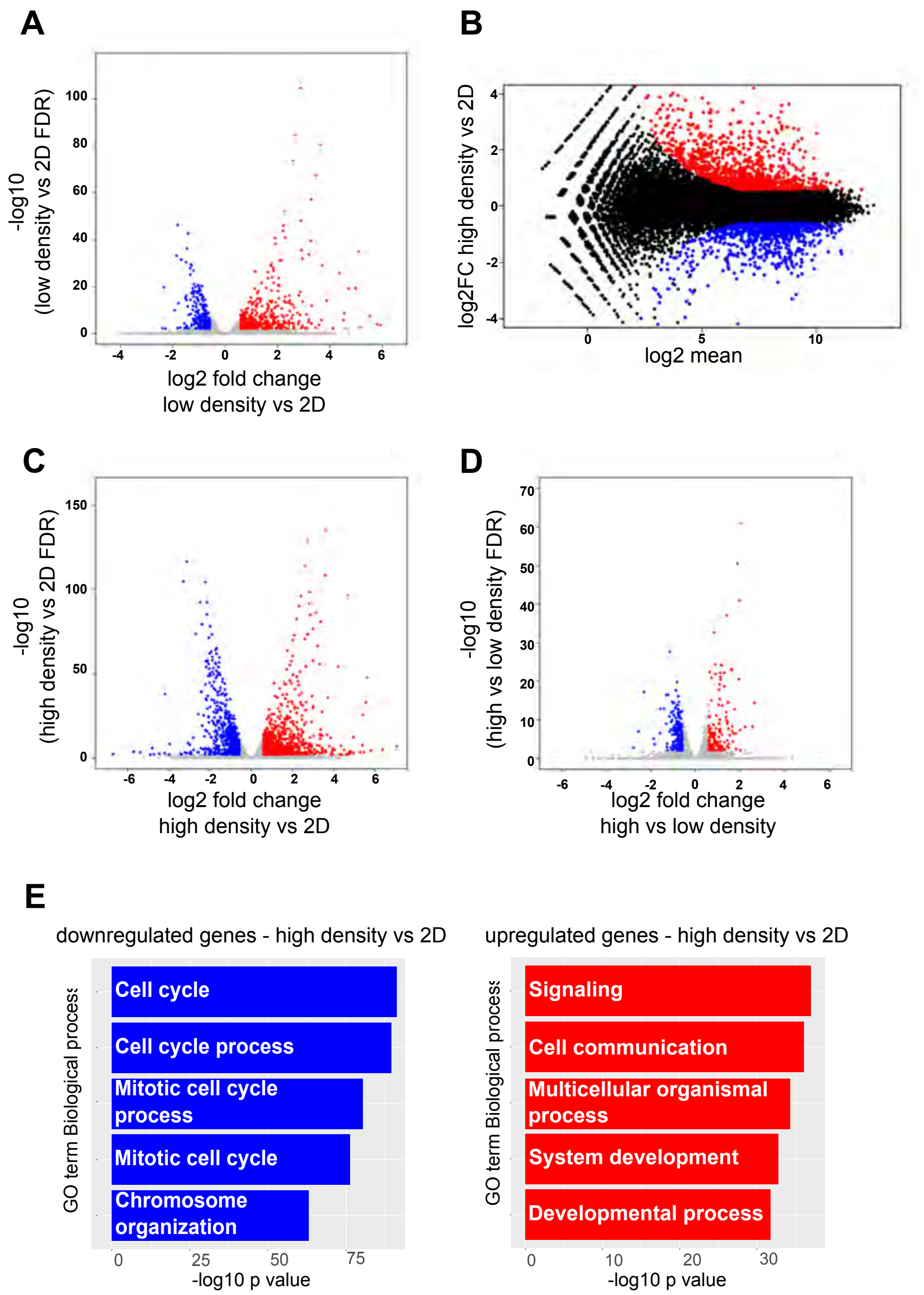

Figure S4. RNAseq data
(A, C-D) Volcano plots illustrating the differentially regulated genes (FDR < 0.01 and fold change > +/- 1.5) between cells cultured in a low-density collagen matrix compared to regular 2D culture (A), high-density collagen matrix compared to regular 2D culture (C), or high-density collagen matrix compared to low-density collagen matrix (D). (B) MA plot illustrating the differentially regulated genes (FDR < 0.01 and fold change > +/- 1.5) between cells cultured in a low-density collagen matrix or in regular 2D culture. (A-D) Genes that are upregulated in are shown in red and downregulated genes are shown in blue. (E) Gene ontology analysis illustrates biological processes most significantly enriched within genes that are upregulated (left panel, red bars) or downregulated (right panel, blue bars) in high-density collagen compared to 2D.

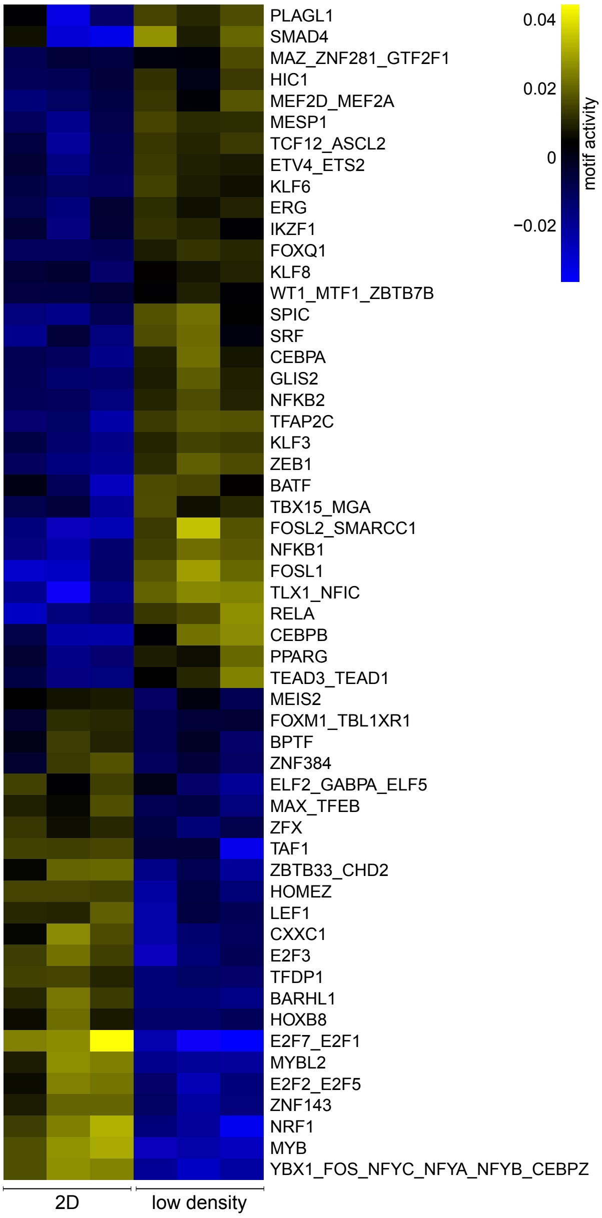

Figure S5. Regulated transcription factors after 2D culture or 3D culture in low density collagen.
Heatmap illustrates the most significantly up- and downregulated TF motifs in low-density collagen vs. regular 2D culture.

Table S2. Sequences of primers used for RT-qPCR. All primers were designed using the NCBI gene database and the primer-BLAST tool.

Gene	Protein	Primer direction	Sequence (5' to 3’)
ACTB	β-actin	Forward	AGAGCTACGAGCTGCCTGAC
		Reverse	AGCACTGTGTTGGCGTACAG
CD69	CD69	Forward	GATGCCACCAGTCCCCATTT
		Reverse	TGGCCCACTGATAAGGCAATG
CD38	CD38	Forward	GGTTTCCCGCAGGTTTGC
		Reverse	TCCACACTCCCAAAAGTGCT
TNFRSF9	C137	Forward	TGCAGGCAGTGTAAAGGTGT
		Reverse	TCGACAGATGCCACGTTTCT
INFG	INFγ	Forward	TCGTTTTGGGTTCTCTTGGCT
		Reverse	TTTTCTGTCACTCTCCTCTTTCCA
TNF	TNFα	Forward	AGCCCATGTTGTAGCAAACCC
		Reverse	GGACCTGGGAGTAGATGAGGT
TGFB1	TGFβ	Forward	CTGGCGATACCTCAGCAACC
		Reverse	GTGAACCCGTTGATGTCCACTT

Table S3. RNA sequencing depth and alignment info

Sample	organism	ref genome	Number of input reads	Uniquely mapped reads	Uniquely mapped reads %
High-density rep1	human	hg19	16316259	12987692	79,60%
High-density rep2	human	hg19	20827452	16565647	79,54%
High-density rep3	human	hg19	15690290	12488770	79,60%
Low-density rep1	human	hg19	17806762	14191720	79,70%
Low-density rep2	human	hg19	14490086	11605314	80,09%
Low-density rep3	human	hg19	11194136	8966651	80,10%
2D rep1	human	hg19	14217463	11462456	80,62%
2D rep2	human	hg19	11640177	9254300	79,50%
2D rep3	human	hg19	12375402	9929856	80,24%
